# Reducing treatment delays of first episode psychosis through policy in Canada: a mixed methods analysis of service provider perspectives

**DOI:** 10.1192/j.eurpsy.2024.795

**Published:** 2024-08-27

**Authors:** F. Poukhovski-Sheremetyev, Y. Pelling, J. Denny, A. Abdel-Baki, S. Iyer, V. Noel

**Affiliations:** ^1^Department of Psychiatry, McGill University; ^2^Douglas Hospital Research Centre, Montréal; ^3^SPOR National Training Entity, Quebec City; ^4^Cape Breton University, Nova Scotia; ^5^Department of Psychiatry and Addictions, University of Montréal, Montréal, Canada

## Abstract

**Introduction:**

Young people with a first episode of psychosis can achieve full remission with prompt treatment. Throughout Canada, early psychosis intervention programs are implementing policies to ensure timely delivery of services. One of Canada’s first early intervention services, the Prevention and Early Intervention for Psychosis program, set the guideline that all youth referred should receive an appointment within 72 hours. The availability of early intervention programs has increased significantly but the standards these programs have adopted to ensure timely delivery of services remains unknown.

**Objectives:**

This project aims to identify the policies and practices in early intervention programs that ensure timely delivery of services. Secondly, the project aims to understand the level of awareness of the 72-hour recommendation and the level of adoption of this recommendation. Thirdly, the project aims to identify the factors that facilitate and hinder a program’s ability to reach and maintain their benchmarks for timely delivery of services.

**Methods:**

Participants included 17 service delivery providers from four early intervention programs located in socio-culturally distinct regions in Canada. Participants completed a survey about their program’s service delivery policies and practices. We led individual semi-structured interviews with seven service providers to identify the barriers and facilitators to delivering timely care. We conducted frequency analyses of the survey data and thematic analysis of the interviews to identify emerging themes.

**Results:**

Forty-one percent of survey respondents indicated that their program implemented formal policies to minimize the delay to the first appointment, with benchmarks ranging from 72 hours to 12 weeks. The majority of program managers interviewed were aware of the 72-hour benchmark, voiced satisfaction with standards, and felt that establishing standards was helpful to delivering quality services. Average time between referral and first appointment ranged from 10 days to 12 weeks; however, more than half of survey respondents were unaware of the average delay in their program. Notable barriers to implementation included patient non-responsiveness, insufficient staffing, and missing patient contact information from referrals. The service providers reported engaged staff, flexible schedules, and team-based care as facilitators to meeting service delivery benchmarks.

**Conclusions:**

Benchmarks such as the 72-hour recommendation are an excellent step in improving timeliness of delivery of early intervention services. Common barriers to meeting benchmarks, such as patient adherence and staff resources may be difficult to overcome; however, implementing standardized referral forms and processes, increasing staff engagement, providing flexible schedules, and encouraging team-based care could improve timely delivery of services.

**Disclosure of Interest:**

None Declared

